# Reaction Modes on a Single Catalytic Particle: Nanoscale Imaging and Micro-Kinetic Modeling

**DOI:** 10.1021/acscatal.2c02901

**Published:** 2022-10-07

**Authors:** Johannes Zeininger, Maximilian Raab, Yuri Suchorski, Sebastian Buhr, Michael Stöger-Pollach, Johannes Bernardi, Günther Rupprechter

**Affiliations:** † Institute of Materials Chemistry, TU Wien, Getreidemarkt 9, 1060Vienna, Austria; ‡ University Service Center for Transmission Electron Microscopy, TU Wien, Wiedner Hauptstraße 8-10, 1040Vienna, Austria

**Keywords:** surface reaction, chemical oscillations, multifrequential oscillations, interfacet communication, coupled oscillators, frequency transformers, field emission microscopy, single-particle imaging

## Abstract

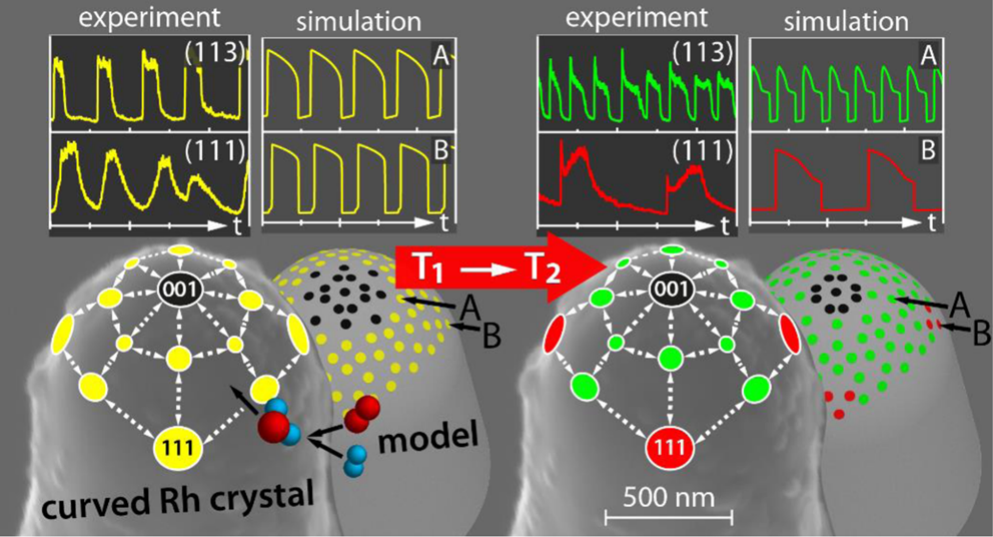

The kinetic behavior of individual Rh(*hkl*) nanofacets coupled in a common reaction system was studied using the apex of a curved rhodium microcrystal (radius of 0.65 μm) as a model of a single catalytic particle and field electron microscopy for in situ imaging of catalytic hydrogen oxidation. Depending on the extent of interfacet coupling via hydrogen diffusion, different oscillating reaction modes were observed including highly unusual multifrequential oscillations: differently oriented nanofacets oscillated with differing frequencies despite their immediate neighborhood. The transitions between different modes were induced by variations in the particle temperature, causing local surface reconstructions, which create locally protruding atomic rows. These atomic rows modified the coupling strength between individual nanofacets and caused the transitions between different oscillating modes. Effects such as entrainment, frequency locking, and reconstruction-induced collapse of spatial coupling were observed. To reveal the origin of the different experimentally observed effects, microkinetic simulations were performed for a network of 105 coupled oscillators, modeling the individual nanofacets communicating via hydrogen surface diffusion. The calculated behavior of the oscillators, the local frequencies, and the varying degree of spatial synchronization describe the experimental observations well.

## Introduction

Metal nanoparticles, frequently used in catalysis, consist of crystallographically differently oriented nanofacets confined by straight edges, sharp corners, or vertices, which are combined in a complex dynamic reaction system. The behavior of such a heterogeneous reaction system depends on the properties of the individual nanofacets, their sizes, and interfacet communication. All these aspects are not accessible by conventional ensemble-averaging methods, thus motivating spatially resolved single particle studies.^1−4^ Particularly the role of interfacet communication in the reaction dynamics is difficult to examine because this requires in situ methods providing local information about the ongoing reaction on several nanofacets simultaneously. Methods that allow such parallel monitoring of a reaction on the individual nanofacets of a nanoparticle used in industrial catalysis have not been developed yet; therefore, proper model systems are required.

Metal nanotips with polyhedral apices have been used as models of individual catalytic particles because the coexistence of crystallographically differently oriented nanofacets is a main property of both, nanoparticles and nanotips.^5^ Such nm-sized polyhedral apices can be characterized in situ with atomic resolution, for example by field ion microscopy (FIM), and the ongoing catalytic reaction can be monitored in situ by FIM,^6,7^ field electron microscopy (FEM),^8−10^ or field desorption microscopy (FDM),^11,12^ including energy analysis of desorbing species at atomic lateral resolution.^13^ These model studies provide an important extension to applied studies by high-resolution transmission/scanning electron microscopy (HRTEM/STEM) on nanoparticles in “powder catalysts”.^14−16^

The edges confining the nanofacets of a nanotip appear to be much more permeable for surface diffusion fluxes of reactants than the grain boundaries confining the domains of a polycrystalline foil. These diffusion fluxes play an important role in the spatial coupling of the reaction on different nanofacets. On a nanotip, diffusive coupling usually results in synchronization of the reaction modes on different facets.^17^ In order to deliberately vary the degree of synchronization between different facets, we introduced a novel model system: a μm-sized curved rhodium crystal, which is an order of magnitude larger than a typical nanotip.^10^ The increase in size extends the diffusion paths and thus attenuates the diffusion-mediated communication between the individual facets, resulting in decoupling effects. Despite its μm-range size, the surface of such a curved crystal can still be imaged with FEM, allowing visualization of reacting species and monitoring of the facet-resolved kinetics and of reaction coupling between facets.^18^

Catalytic H_2_ oxidation belongs to the reactions which can be visualized in FEM due to the sufficient image contrast between the catalytically inactive Rh surface (high O_ads_ coverage, high work function, and dark contrast) and the active surface (low H_ads_ and O_ads_ coverage and bright contrast due to the lower work function).^19^ Resulting from a significant difference in diffusivity of hydrogen and oxygen on rhodium surfaces (oxygen can be treated as immobile compared to hydrogen at usual reaction conditions), H_2_ oxidation and particularly its oscillating mode are proper targets for studying the role of spatial coupling and synchronization. Even more so as already a mesoscopic model system of coupled μm-sized domains of a polycrystalline metal foil revealed features that have not been observed before: multifrequential oscillations,^20^ frequency transforming by grain boundaries,^21^ and simultaneously existing multistates.^22^

On the nm scale, such effects have not been detected until recently: oscillating modes of catalytic reactions, such as H_2_ or CO oxidation or NO reduction on Pt, Pd, or Rh nanotips, have always shown a synchronized behavior over the whole nanocrystal surface.^6−8,17^ In contrast, the use of a μm-sized curved crystal with significantly larger facets (size of 10–100 nm) provided novel insights: in a recent short communication, we reported the first observation of the desynchronization of oscillations in catalytic H_2_ oxidation on a curved Rh microcrystal.^18^ In situ FEM monitoring has shown that the reaction simultaneously oscillated with different frequencies on adjacent Rh(*hkl*) nanofacets having differing crystallographic structures. This is quite unusual because the surface facets on the apex of a curved crystal are separated solely by interfacet edges and not by grain boundaries having a drastic influence on the diffusion fluxes. Apart from multifrequential oscillations, entrainment, frequency-locking, limited interfacet coupling, and collapse of spatial coupling were observed on the curved microcrystal in this reaction.^18^

Herein, we significantly extend and further deepen these insights by focusing on coupling and desynchronization of the reaction process on individual facets of a single catalytic particle. To rationalize the present experimental observations, microkinetic simulations of hydrogen oxidation were performed for a network of 105 individual nanofacets acting as individual oscillators coupled in a spatial grid mimicking the curved crystal surface. Varying the degree of the spatial coupling, all different modes of the oscillating reaction behavior observed in the experiments were reproduced with a high degree of quantitative agreement.

## Crystallographic Layout of the μm-Sized Curved Rhodium Crystal

**Figure 1:**
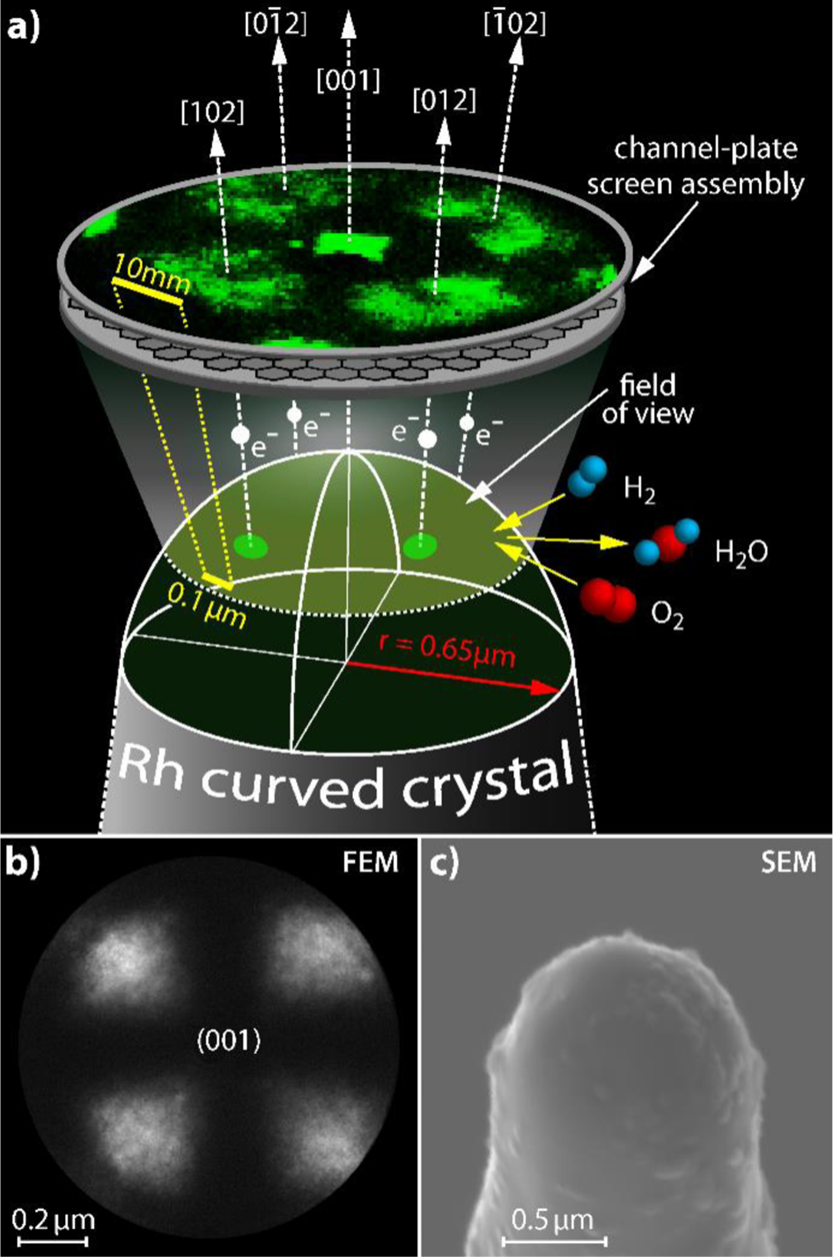
(a) Principle of FEM imaging: electrons, field-emitted from the sample surface, create a magnified projection image of the apex surface on the screen. (b) FEM micrograph of the clean Rh surface. The fourfold symmetry indicates the [001]-orientation. (c) SEM micrograph of the Rh microcrystal with a hemispherical apex with a radius of 0.65 μm.

The Rh microcrystal was fabricated by repetitive electrochemical etching of a Rh wire (0.1 mm, MaTeck, 99.99%). The tip was shaped, and its surface was cleaned by field evaporation in UHV using FIM control with Ne^+^ ions for imaging. Finally, the resulting hemispherical apex of the Rh tip was blunted by annealing to 1300 K, forming the curved crystal (Figure 1).

Experiments were performed in an ultra-high vacuum (UHV) apparatus consisting of the FEM/FIM chamber, including a sample holder which allows operation in a controlled temperature range of 78–900 K, a channel-plate/screen assembly, and a gas supply system. The FEM/FIM chamber was used as a gradient-free flow reactor in which the Rh microcrystal was exposed to gaseous molecular oxygen and hydrogen in the 10^–6^ mbar pressure range, while the ongoing catalytic H_2_ oxidation was visualized in situ by FEM. In FEM, the potential barrier on the surface is deformed by an applied electric field, leading to the tunneling of electrons into a vacuum, where they form a projection image on a screen with a magnification of ∼10^6^ and a resolution of 2 nm (Figure 1a,b). A CCD camera (Hamamatsu C13440) was used for in situ recording of FEM images, the temperature of the sample was measured by a Ni/CrNi thermocouple directly spot-welded to the shaft of the microcrystal.

The image contrast in an FEM is provided by differences in the local work function, which are convoluted with the local electrostatic field. As a result, adsorbed reactants can be directly identified on the sample surface, provided the work function differences are sufficient for discernible image contrast.^8−10^ In addition to “static” imaging by FEM, dynamic surface processes can be visualized in real-time, such as diffusion^23^ or catalytic surface reactions.^5,8−10^ Because both the local work function and the local catalytic activity depend on the surface concentrations of reactants, the variation of the local FEM image intensity reflects the catalytic activity (kinetics by imaging^24^).

The reactant gases (O_2_ and H_2_) were dosed by precision leak valves, and the gas phase composition was verified by a residual gas analyzer (QMS: MKS e-Vision 2). The reaction product H_2_O was continuously pumped off by a turbomolecular pump.

**Figure 2:**
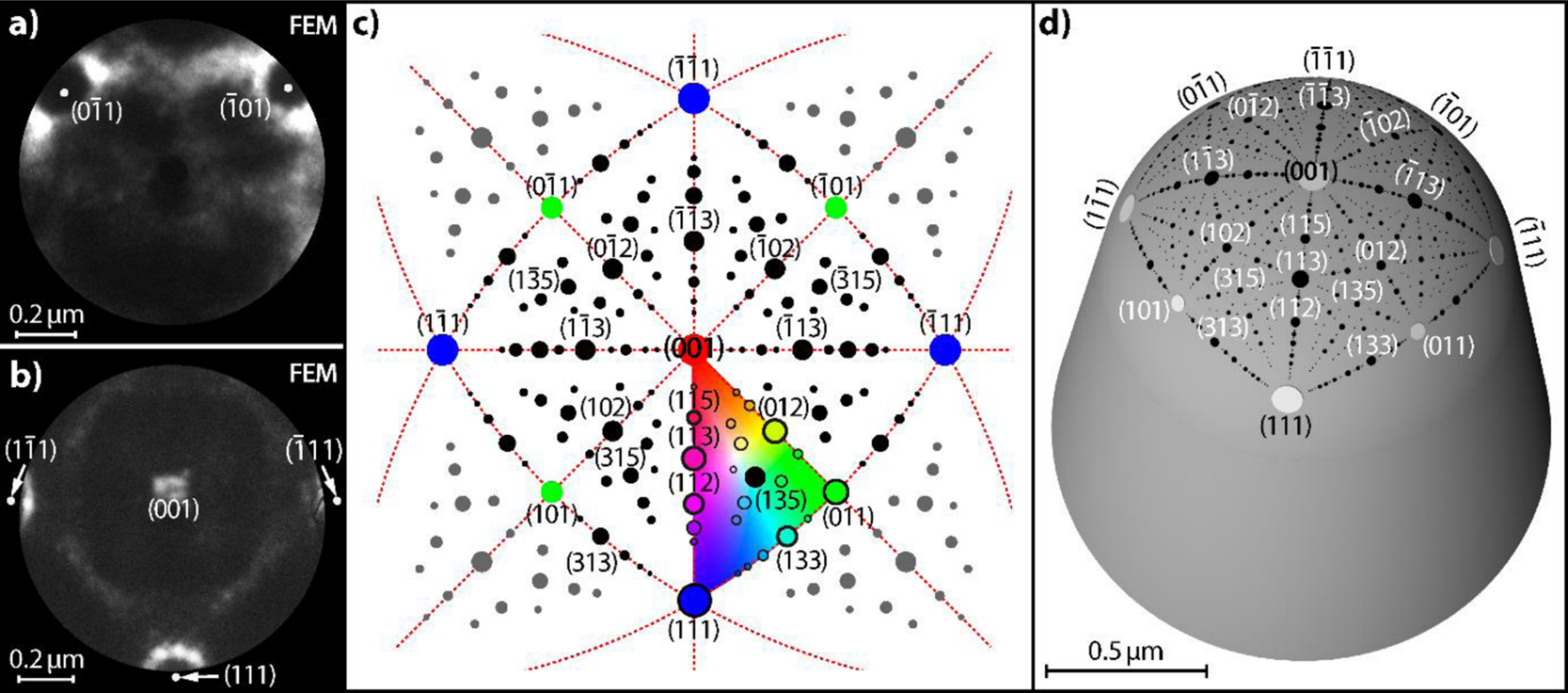
Determination of the crystallographic layout of the curved Rh crystal surface: (a) FEM micrograph of the Rh surface after CO treatment at *T* = 600 K; the {011} facets are surrounded by residual carbon layers (bright “collars”); (b) in situ FEM micrograph taken during the ongoing H_2_ oxidation at *T* = 453 K. The catalytically active lower (111) facet and bright regions surrounding two lateral facets of this type are visible; (c) corresponding [001]-oriented fcc stereographic projection, the colored triangle corresponds to the inverse pole figure including all possible crystallographic orientations; (d) 3D model of the curved Rh crystal with the crystallographic net as the overlayer.

In the case of a μm-sized curved Rh crystal, the precise determination of its crystallographic layout represents an experimental challenge: the usual direct assignment of the crystallographic structure based on FIM imaging with atomic resolution does not work in this case because the radius of the crystal apex would require FIM voltages unrealistic for the present setup. Therefore, the sample crystallography was determined by combining symmetry arguments, deduced from FEM micrographs (Figure 2a,b), for which the required FEM voltages were much lower than those for FIM, with the stereographic projection. The radius of the crystal apex (0.65 μm, Figure 1c) was determined by SEM (FEI Quanta FEG 250).

Figure 1b shows the clean Rh surface imaged by FEM in UHV, with the fourfold symmetry indicating the (001)-orientation of the central facet. To determine the exact positions of Rh{011} facets, the specimen was exposed to CO at *p*_CO_ = 2.3 × 10^–6^ mbar and *T* = 600 K. According to literature, CO dissociates under these conditions on the Rh surface leading to the formation of carbon crystallites around the Rh{011} facets.^25^ This effect, visible in Figure 2a as bright “collars” confining the dark spots, allows locating the {011} facets. In turn, a snapshot taken during ongoing H_2_ oxidation at *p*_O_2__ = 4.4 × 10^–6^ mbar, *p*_H_2__ = 4.8 × 10^–6^ mbar, and *T* = 453 K allows for the identification of three catalytically active {111} facets encircled by bright rings, partially visible in Figure 2b (the fourth facet is outside the field of view). Figure 2c displays the (001)-centered fcc stereographic projection oriented in accordance with the symmetry axes in Figure 2a. The stereographic projection allows the indication of the remaining facets in the field of view. A corresponding 3D model of the present curved crystal with the overlayer showing the relative positions of crystallographically different facets is presented in Figure 2d. The circular field of view visible in FEM has a diameter of about 1.1 μm. Based on the known crystallographic layout and the SEM-determined shape of the crystal apex, atomic ball models of particular surface regions can be constructed, as will be shown below.

## Facet-Resolved Hydrogen Oxidation on Rhodium

On platinum group metals, catalytic hydrogen oxidation follows the Langmuir–Hinshelwood mechanism: the reactants hydrogen and oxygen dissociatively adsorb on neighboring surface sites prior to reaction, and the product water desorbs at the used reaction temperatures.^26^ In oxygen excess, when the catalyst surface is covered by O_ads_, the reaction system is in a steady state of low catalytic activity. This changes via a kinetic transition to high catalytic activity in an excess of hydrogen sufficient to switch the competitive H/O coadsorption in favor of hydrogen. Kinetic transitions exhibit a hysteresis caused by the different adsorption properties of hydrogen and oxygen, further depending on the metal and crystallographic surface orientation.^27^

Apart from these steady states, under particular conditions the H_2_ oxidation reaction on Pt, Pd, or Rh may oscillate between the active and inactive states in a self-sustained way, that is, the reaction rate may vary periodically despite constant external parameters such as the temperature, pressure of reactants, and so forth.^28−30^ In comparison to other oscillating surface reactions, such as CO oxidation^30,31^ or NO reduction,^7,32^ studies of oscillating H_2_ oxidation are still rather scarce, despite the important involvement of this reaction in the hydrogen conversion in fuel cells,^33^ catalytic heat production,^34^ elimination of hydrogen via catalytic recombination,^35^ and hydrogen sensors.^36^

**Figure 3:**
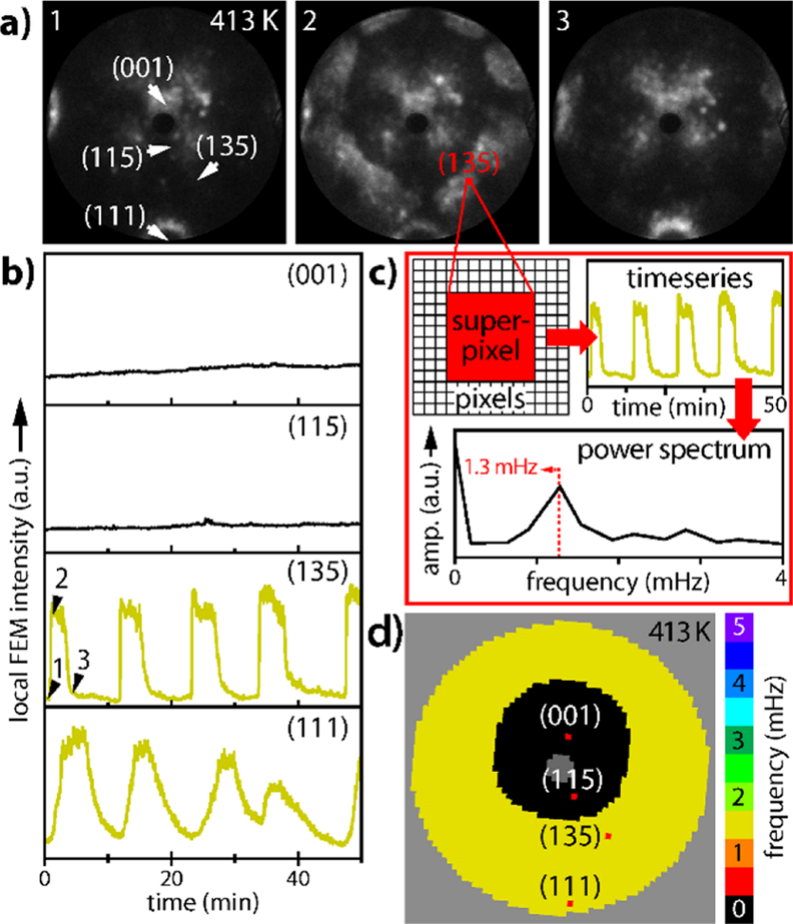
Synchronized oscillations in the H_2_ oxidation reaction on the curved Rh crystal at *p*_O_2__ = 4.4 × 10^–6^ mbar, *p*_H_2__ = 4.8 × 10^–6^ mbar, and *T* = 413 K: (a) in situ FEM video-frames (diameter of the circular field of view 1.1 μm); (b) time series recorded as local FEM intensity for the nanofacets marked in frame 1. Time points 1 to 3 correspond to the frames in (a); (c) construction of the frequency map. Local FEM intensity values read out for superpixels (8 × 8 pixels of the recorded image) provide the set of time series. The power spectra of the time series calculated by fast Fourier transformation provide the frequency values; (d) calculated frequencies for all superpixels form the color-coded frequency map.

In the present study, self-sustained oscillations in H_2_ oxidation on the curved Rh crystal were observed within the 10^–6^ to 10^–5^ mbar pressure range for temperatures from 413 to 453 K. At constant partial pressures of *p*_O_2__ = 4.4 × 10^–6^ mbar, *p*_H_2__ = 4.8 × 10^–6^ mbar and constant temperature of *T* = 413 K (Figure 3), oscillations occur in a synchronized way almost over the whole sample apex. Increasing the *p*_H_2__/*p*_O_2__ ratio leads to a collapse of oscillations and, eventually, a transition to the catalytically active state is observed.^22^ Acquisition of in situ FEM video-footage (Figure 3a) demonstrates this via FEM intensity curves locally analyzed for regions of interest (ROIs, corresponding to 20 × 20 nm^2^ of the surface area) placed on Rh(135), Rh(111), Rh(115), and Rh(001) facets, as displayed in Figure 3b.

The parallel imaging principle of FEM (all surface sites emit electrons simultaneously) allows monitoring in parallel the intensity of each of the (448 × 448) pixels and thus enables the construction of a frequency map. For practical reasons, a binning into superpixels (8 × 8 pixels, corresponding to 20 × 20 nm^2^) was applied, resulting in 2182 superpixels for the entire field of view. The local evolution of the FEM intensity is then extracted from the video file as a time series for each superpixel, exemplarily shown in Figure 3b for four superpixels placed on the different Rh(*hkl*) facets. The signal is converted from the time domain to the frequency domain by using a fast Fourier transformation. From the location of the strongest peak in the power spectrum (Figure 3c), the main frequency can be determined. These obtained main frequency values are plotted into a grid resulting in a color-coded frequency map (Figure 3d). In this way, spatial correlations and differences in local frequencies of oscillating regions are visualized. Because the positions of particular Rh(*hkl*) facets are known, the observed phenomena can be directly related to the crystallographic layout of the sample surface.

The frequency map in Figure 3d convincingly confirms that the oscillations observed at the given conditions are synchronized across the whole apex surface except for a (nonoscillating) circular central region with a diameter of about 430 nm.

This behavior is in line with previous nm-scale observations of the oscillating hydrogen oxidation: kinetic transitions between the catalytically active and inactive steady states on nanotips always occurred spatially synchronized over the entire apex surface.^8,10^ Such global synchronization must result from coupling provided by the surface diffusion of hydrogen because gas phase coupling can be neglected under high vacuum conditions. An analogous degree of synchronization on the nm scale was observed in CO oxidation and NO reduction reactions. This led to the general conception that self-sustaining oscillations (and generally kinetic transitions) in catalytic nano-systems occur spatially synchronized. However, this conception fails under certain conditions, as described in the following.

**Figure 4:**
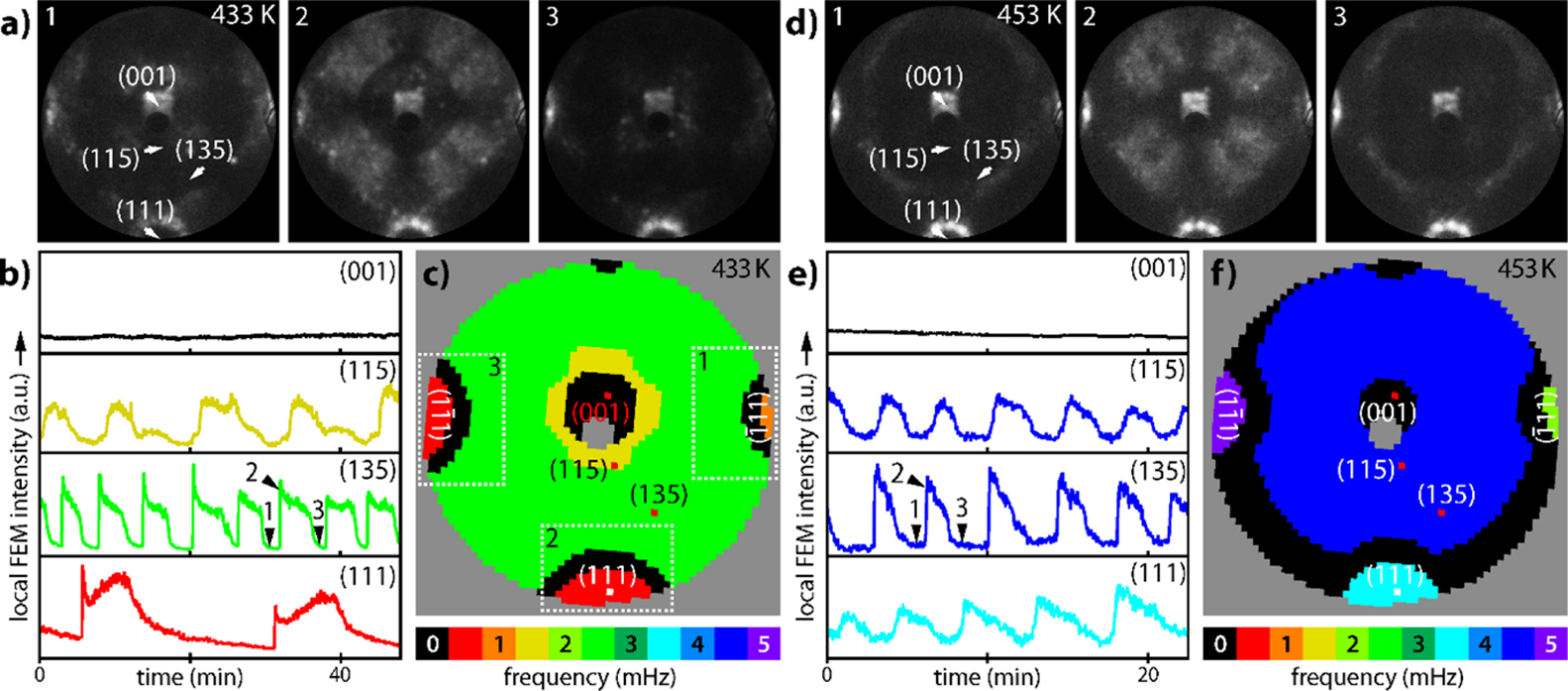
Multifrequential self-sustained oscillations in the catalytic H_2_ oxidation reaction on the curved Rh crystal; (a–c) oscillating behavior at *p*_O_2__ = 4.4 × 10^–6^ mbar, *p*_H_2__ = 4.8 × 10^–6^ mbar, and *T* = 433 K; (a) in situ FEM video frames; (b) time series recorded as local FEM intensity for the nanofacets marked in frame 1. Time points 1 to 3 correspond to the frames in (a); (c) corresponding color-coded frequency map; (d–f) same but at 453 K.

The behavior of the present system drastically changes at higher sample temperature: at 433 K, local *multifrequential* oscillations appear (Figure 4), where the peripheral {111} regions (red and orange in the frequency map of Figure 4c) oscillate fully detached from the surrounding region (green in the frequency map), although they are separated solely *by tens of nanometers*.

The FEM intensity curves in Figure 4b illustrate the discrepancies in the frequencies for different regions, which differ by a factor of 3.8. The appearance of the multifrequential oscillations is accompanied by a contraction of the central nonoscillating region. The “new” oscillating part of the previously nonoscillating central region (yellow in the frequency map of Figure 4c) exhibits a double-period characteristic. This phenomenon is caused by the interplay between the periodic reaction fronts entering the {115} regions from outside and the local formation rate of subsurface oxygen. Due to the local rate of subsurface oxygen formation,^37^ the time interval necessary to replenish the subsurface oxygen reservoirs of the {115} regions is longer than the period of the “forcing” fronts; thus, only each second front propagation can be carried forward, resulting in a period doubling. Period doubling is a form of frequency locking, a phenomenon observed when a system or a region oscillates with a frequency different from its natural frequency due to forcing by an external stimulus.^38^ A similar period doubling was previously observed in CO oxidation on a Pt(110) single crystal,^39^ but not yet detected on the nanoscale.

Further increasing the temperature from 433 to 453 K again changes the system behavior: the double-period region (yellow in Figure 4c) synchronizes with its surrounding (the blue region in Figure 4f). The latter contracts and changes the frequency from 2.7 to 4.3 mHz (compare Figure 4b,e). The {111} regions on the periphery of the imaged area continue to oscillate with their own frequencies, different from the rest of the sample and each other (Figure 4f).

This is very surprising because the distance between any two sample facets is smaller than the surface diffusion length of hydrogen, which is in the order of μm under the present conditions. Consequently, the oscillations on the different nanofacets should be synchronized by coupling via surface diffusion. As mentioned, such synchronized oscillations were so far considered an established property of nanosystems, a widespread opinion, which must be revised now.

**Figure 5:**
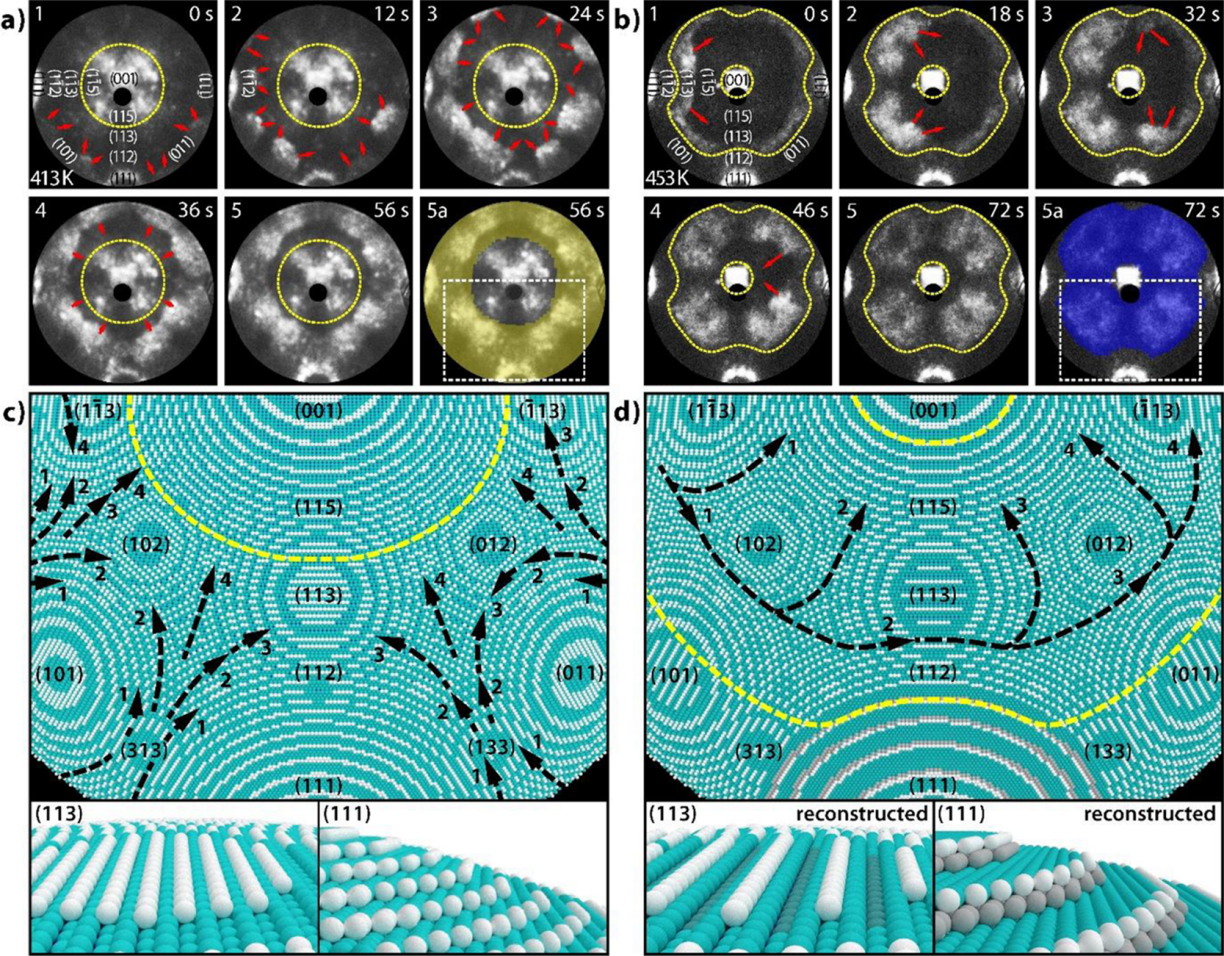
Reaction front propagation in H_2_ oxidation on the curved Rh crystal at constant *p*_O_2__ = 4.4 × 10^–6^ mbar and *p*_H_2__ = 4.8 × 10^–6^ mbar: (a) kinetic transition from the inactive to the active state at 413 K illustrated by FEM video frames with enhanced image contrast for better visibility. Frame a shows frame 5 overlayed with the frequency map from Figure 3d; (b) same as in (a), but at 453 K. Frame a shows frame 5 overlayed with Figure 4f; (c) surface structure model (*r*_tip_ = 100 nm) of the region marked by a white dashed rectangle in the frame (a). The black arrows show the direction of the propagating reaction fronts; the yellow dotted line frames the area of the reaction front propagation. The arrow numbers indicate the state of the front propagation corresponding to the FEM video frames. Lower insets: unreconstructed surface of the (113) and (111) facets; (d) same as in (c), but for the reconstructed surfaces.

It seems that under the present conditions, the spatial coupling partially collapses, although obstacles known to cause this, such as grain boundaries between the domains of a polycrystalline foil,^20,21^ are absent in the present sample. The regions oscillating with different frequencies in the present study are confined merely by “nano ridges” and highly stepped surfaces between the facets. In the present experiments, we observe two types of temperature-caused changes: (i) the oscillating behavior changes qualitatively between 413 and 433 K; (ii) rather quantitative changes take place between 433 and 453 K. Consequently, the responsible surface processes for the partial collapse of coupling may occur between 413 and 433 K. According to previous studies, in this temperature interval, Rh{011} and Rh{113} facets undergo a (1 × 2) missing-row reconstruction,^40−42^ and the step-doubling reconstruction on the [111]-oriented vicinal Rh surfaces occurs.^43^ Thus, the reaction proceeds on surfaces of different crystallography at 433 K than at 413 K (see the corresponding ball models in Figure 5). This leads to a differing spatial evolution of the reaction: changes in surface crystallography may reduce the local hydrogen diffusion and alter the propagation of reaction fronts, even impeding their spreading to certain areas. As these reaction fronts are responsible for transporting the oscillation frequencies within coupled areas, the extent of the synchronized surface processes changes. The reconstructions create protruding atomic rows, which effectively block the propagation of fronts and *decouple* the central oscillating region from the peripheric {111} areas (Figure 5). In other words, the protruding atomic rows play the role of local hydrogen diffusion barriers and therefore serve as nanosized frequency transformers.

The reconstructions lead to remarkable changes in the reaction front propagation during the oscillations in the catalytic hydrogen oxidation, as illustrated in Figure 5. Figure 5a shows the propagation of reaction fronts at 413 K. The fronts start outside the field of view, propagate toward the (001) region, and eventually merge. The front propagation area is equal to the synchronized region in the corresponding frequency map (cf. Figure 3d), as visualized by the overlay of the frequency map from Figure 3d with the video frame at 56 s in Figure 5a.

At 453 K, the surface crystallography is altered by reconstructions, leading to the different behavior of the reaction front shown in Figure 5b. A kinetic transition during the self-sustained oscillations at this temperature is shown as a set of FEM video frames with red arrows indicating the direction of the front propagation. The transition starts with a reaction front nucleation on the Rh(11̅2) facet acting as the pacemaker. The front then spreads along a circle-like path, surrounding the central region in a pincer movement and merging eventually, without progressing into the enclosing regions (outside the yellow dotted lines). The whole surface area, where the reaction fronts propagate, is synchronized and exhibits the same oscillation frequency, as visualized by the overlay with the frequency map in Figure 5b (frame 5a). The front propagation for both temperatures is illustrated with ball models in Figure 5c,d, with the ball model for 453 K showing the reconstructed surface, as well as a detailed comparison of the reconstruction-induced changes of the {113} and {111} facets. At 453 K, the extent of front propagation is shifted closer toward the (001) facet than at 413 K; however, the front still cannot proceed into the vicinal (001) regions. On the reconstructed surface, the formed double steps surrounding the {111} vicinal facets are aligned in series and in this way amplify their hindering effect on the front propagation toward the {111} facets, thereby effectively decoupling them.

**Figure 6:**
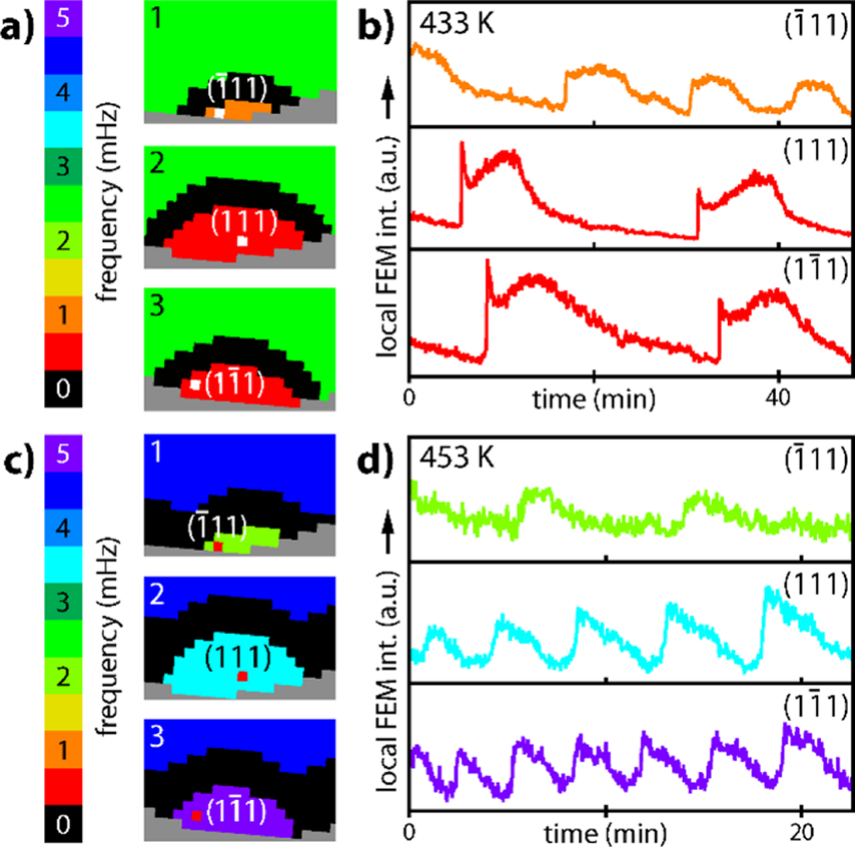
Multifrequential oscillations on Rh{111} facets at *p*_O_2__ = 4.4 × 10^–6^ mbar and *p*_H_2__ = 4.8 × 10^–6^ mbar: (a) *T* = 433 K. Fragments of the frequency map from Figure 5c; (b) corresponding time series recorded as local FEM intensity of superpixels placed on the (1̅11), (111), and (11̅1) facets; (c,d) same as in (a,b), but at *T* = 453 K.

Altogether, this results in the emergence of multifrequential oscillations on different {111} nanofacets with each of them oscillating with its own frequency. At first glance, this seems to be surprising: the oscillation frequency in H_2_ oxidation on Rh is unambiguously related to the surface structure via the activation energy of subsurface oxygen formation.^18,20−22^ This means that the natural local frequency of the three {111} nanofacets visible in the field of view should be the same even without diffusive coupling by hydrogen diffusion. However, all three regions around the facets of the same {111} orientation oscillate with different frequencies differing by factors of up to 1.9 (at 433 K) and 2.3 (at 453 K) for the “fastest” and “slowest” facet, respectively (Figure 6, see also the frequency maps in Figure 4c,f).

This highly unusual effect can be explained by the local peculiarities in the surface relief of the respective regions differing slightly at the atomic level. Presumably, the annealed curved crystal shows slight deviations in the size of the {111} facets, and thereby the quantity of terraces and the atomistic shape of the steps, which leads to the deviations in the rate of subsurface oxygen formation at 433 K. Because the latter is governing the oscillation frequency, it may differ for slightly differing {111} facets as well. Local surface rearrangement at *T* = 453 K can additionally form inhomogeneities, which can act as local pacemakers, whose frequency is imposed on the corresponding region. Such effect is known to contribute to the differences in the local frequency.^44^

## Micro-kinetic Modeling

To rationalize the main experimental finding, namely the transition from monofrequential to multifrequential oscillations, microkinetic modeling was performed. The general model was originally introduced by McEwen et al.,^45^ to simulate field-induced oscillations, observed in an FIM, where the high electric field of >10 V/nm has significantly reduced the activation barrier for oxygen incorporation. The present studies were performed by FEM, where the applied field is much lower (<5 V/nm) and, more importantly, in the opposite direction. The field direction away from the sample surface prevents field-induced changes of the electron density near the surface and thus the field-induced modification of adsorption energetics:^46^ field emission of electrons sets in long before field-induced changes can occur. The absence of field effects on surface reactions was directly proven by pulsed-field supply with varying duty pulses for CO oxidation^46^ and was discussed in detail for H_2_ oxidation.^27^ Besides, the pulsed field supply also proves the absence of the emission current effect, which is also not expected, due to its nA range. Because field effects can be neglected in the present case, the field-dependent terms were omitted in the current modeling. Such a field-free model was used in our previous nano- and mesoscale studies to explain the dependence of the oscillating behavior of H_2_ oxidation on Rh on the activation energy of subsurface oxygen formation.^10,18,20,22^ However, such a simplified one-oscillator model cannot mimic the interfacet coupling effects observed in the present study. Therefore, we extended the original model to a network of 105 oscillators, each simulating a nm-sized facet on the tip surface, coupled by surface diffusion of hydrogen.

**Figure 7:**
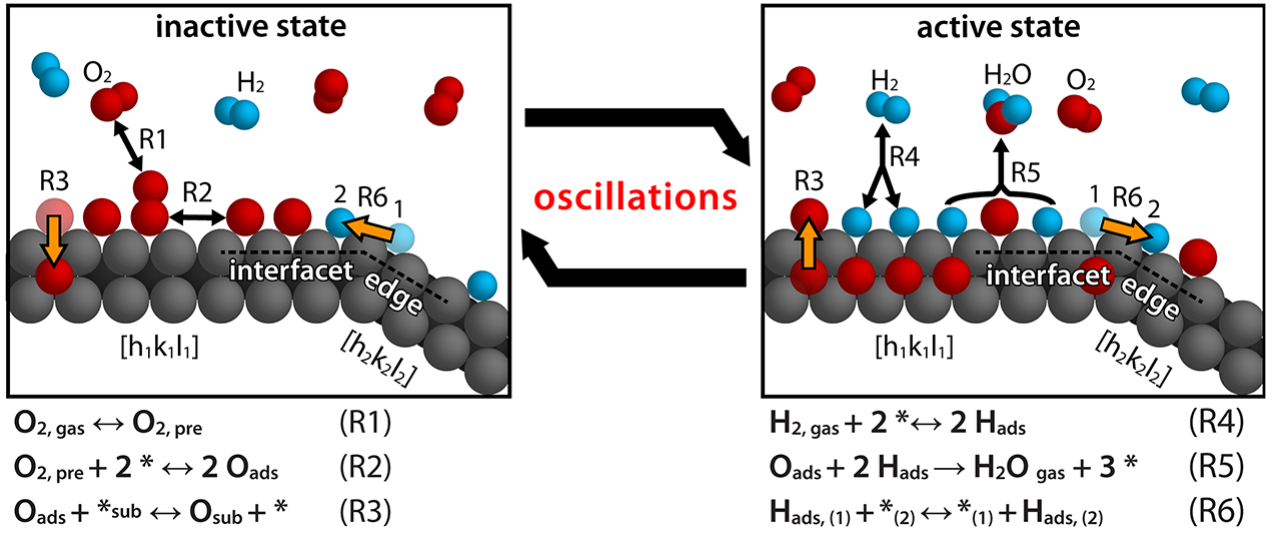
Illustration of the mechanism of self-sustaining oscillations in catalytic hydrogen oxidation on Rh. Atoms are colored: Rh (grey), O (red), and H (blue). The corresponding reaction eqs R1–R6 are listed below, (*) and (*_sub_) represent empty surface and subsurface sites, respectively.

The model is based on the Langmuir–Hinshelwood reaction mechanism with the formation and depletion of subsurface oxygen as the feedback mechanism governing the oscillation cycle. The latter can be summarized as follows: on the rhodium surface, hydrogen and oxygen adsorb competitively, whereby the partial pressures and the temperature decide the “winner”. Oxygen adsorption occurs via a molecular precursor (Figure 7 R1), followed by dissociation into two chemisorbed oxygen atoms (Figure 7 R2). In the oxygen-covered, catalytically inactive state, adsorbed oxygen atoms may migrate to subsurface positions, preferentially at step edges and kink sites,^22,37^ forming subsurface oxygen (Figure 7 R3). The presence of subsurface oxygen reduces the sticking coefficient of oxygen, causing, under proper pressure conditions, favored dissociative adsorption of hydrogen (Figure 7 R4). This switches the system to its active state, with only low surface coverages of both hydrogen and oxygen due to the continuous catalytic water formation, with the product immediately desorbing from the surface (Figure 7 R5) under the given reaction conditions.^26^ The formation of OH intermediates is not considered in the model.

The low surface coverage of oxygen in the catalytically active state leads to a gradient of the chemical potential of oxygen, which drives subsurface oxygen atoms to the surface, where they are consumed in the reaction. As the subsurface oxygen depot, therefore, empties, the sticking coefficient of oxygen recovers, leading eventually again to preferential adsorption of oxygen from the gas phase. The reaction switches back to the inactive state, thereby closing the oscillation cycle.^10,18,20−22^

On a heterogeneous surface, the spatio-temporal evolution of a catalytic reaction depends on the coupling between adjacent surface regions, which occurs via the surface diffusion of reactants^10,17^ or via the gas phase.^47^ Under the present conditions, at which the diffusion of oxygen is very slow and can therefore be neglected,^46,48^ coupling occurs via surface diffusion of hydrogen.^49^ In our model, we used *E*_dif_^H^ = 0.187 eV, adapted from refs (48) and (49); however, similar results are obtained with different values within the range from 0.118 to 0.216 eV reported in the literature.^48−52^ This is taken into account via a diffusion term describing the exchange of hydrogen between adjacent surface regions (Figure 7 R6).

The apex of the curved Rh crystal exhibits a collection of such surface regions (facets of 10 to 100 nm in diameter) with, due to varying crystallography, different properties, for example sticking coefficient of oxygen and the activation energy for the formation of subsurface oxygen. In the present model, this collection is represented by a network of 105 individual oscillators connected to each other.

**Figure 8:**
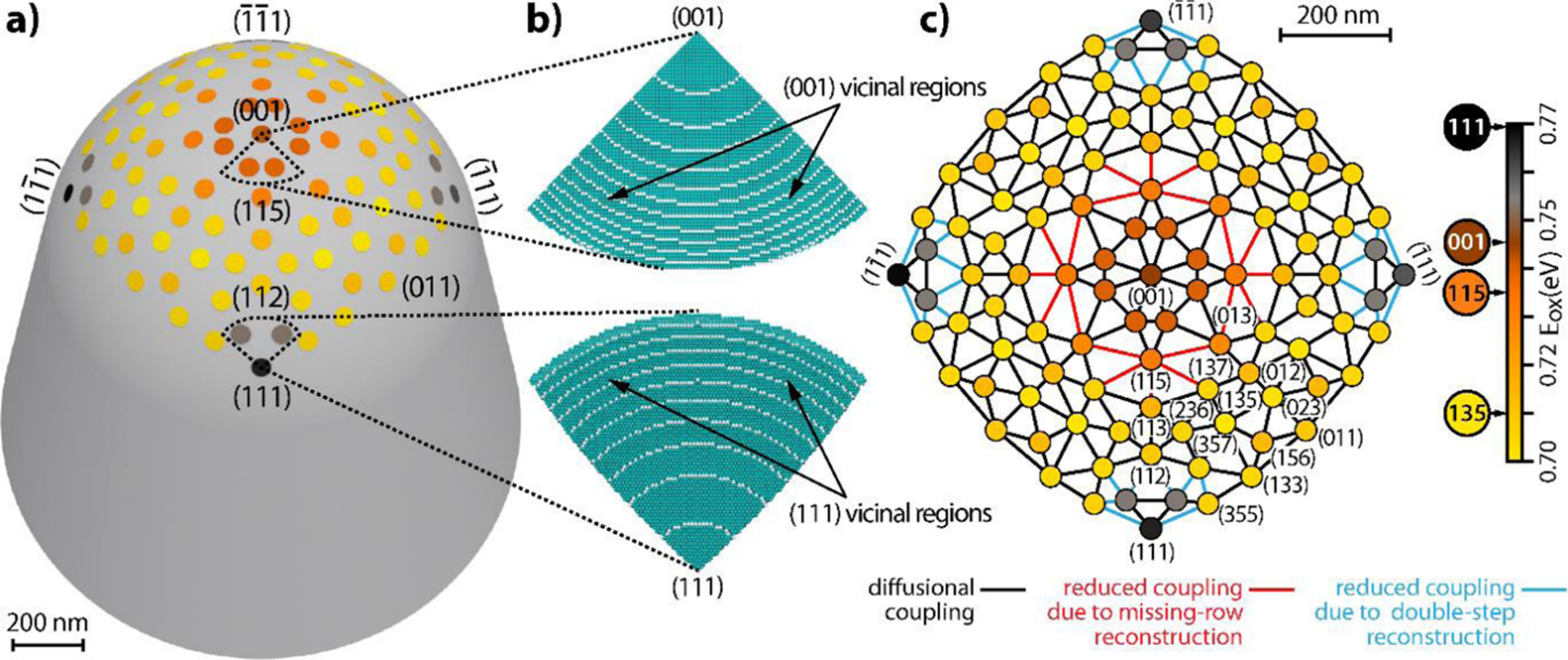
Layout of the 105-oscillators grid for micro-kinetic network modeling: (a) schematic drawing of the curved Rh crystal with the oscillators as an overlayer; (b) ball models of the sectors framed with dashed lines in (a); (c) layout of the oscillator grid. The individual oscillators are color-coded according to their effective activation energies for subsurface oxygen formation. The network of lines indicates the coupling via hydrogen diffusion. The red and blue lines represent reduced diffusion coupling at 433 K due to the missing-row reconstruction and due to the step-doubling reconstruction on the (111)-oriented terraces of vicinal Rh surfaces, respectively.

The network is based on the crystallographic layout of the curved Rh crystal, as illustrated in Figure 8a. Each oscillator is assigned either a certain (*hkl*) orientation or the affiliation to the stepped vicinal regions around the (001) and (111) facets (Figure 8b). The coupling lines connecting the individual oscillators form the oscillator network (Figure 8c).

In this multioscillator model, the hydrogen coverage *θ*_*H*_^*i*^, the oxygen coverage *θ_O_^i^*, and the subsurface oxygen coverage *θ*_*S*_^*i*^ of each individual oscillator are described by the following kinetic equations
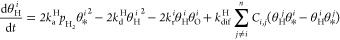

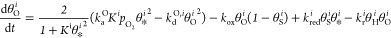

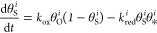
where *i* is the index of the respective oscillator, and θ_*_^i^ represents the empty surface sites defined as θ_*_^i^ = 1 – θ_H_^*i*^ – θ_O_^*i*^. The rate and coupling constants are given by
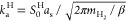

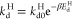

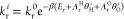

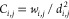

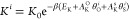

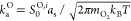

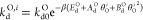

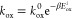

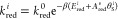
where *S*_0_^H^ is the initial sticking coefficient of hydrogen, *a*_s_ denotes the area of a surface site (10 Å^2^), *m*_H_2__ corresponds to the average molecular mass of hydrogen, and β = *k*_B_*T*. The parameters *S*_0_^O,*i*^ and *m*_O_2__ represent the local initial sticking coefficient of oxygen and its molecular mass, respectively.

The diffusive coupling by hydrogen is considered by the *C*_*i*,*j*_, the distance-weighted coupling coefficient between oscillators *i* and *j*, and *d*_*i*,*j*_, the distance between the respective oscillators. The *w*_*i*,*j*_ values are coupling factors, generally equal to 1 (corresponding to black lines in Figure 8c). The (1 × 2) missing-row reconstruction on Rh{011} and Rh{113} facets and step-doubling reconstruction on the (111)-oriented terraces of vicinal Rh surfaces, occurring in the temperature interval between 413 and 433 K,^40−43^ form diffusion barriers which limit the reaction front propagation.^18^ This is incorporated into the model by reduced hydrogen coupling factors across the reconstructed surface zones in the simulations for 433 K: red lines in the network (Figure 8c) for reduced coupling due to missing-row reconstruction (*w*_mr_) and blue lines for reduced coupling due to the step-doubling reconstruction (*w*_sd_). The coupling factors have been fitted to match the temperature-dependent desynchronization behavior. The initial sticking coefficients and the other kinetic parameters used in the present calculations are adapted from refs (45) and (48) and are listed in Table 1.

**Table 1:** Parameters Used in the Micro-Kinetic Model Simulations of the Coupled Oscillator Network^a^

symbol	parameter description	value
*S*_0_^H^	initial sticking coefficient of H	0.3
*k*_d0_^h^	prefactor for hydrogen desorption	3.0 × 10^10^
*E*_d_^H^	desorption energy of H	0.6
*k*_r_^0^	prefactor for water formation	7.0 × 10^12^
*E*_r_	activation energy for water formation	0.79
*A*_r_^H^	coverage dependence of the activation energy of water formation on H	–0.27
*A*_r_^O^	coverage dependence of the activation energy of water formation on O	–0.145
*k*_dif0_^h^	prefactor for hydrogen diffusion	1.5 × 10^7^
*E*_dif_^H^	activation energy for hydrogen diffusion	0.187
*K*_0_	prefactor for the oxygen dissociation equilibrium constant	0.2525
*E*_*k*_	activation energy for the oxygen dissociation equilibrium constant	–0.178
*A*_K_^O^	coverage dependence of adsorbed oxygen on oxygen dissociation	0.158
*A*_K_^s^	coverage dependence of subsurface oxygen on oxygen dissociation	0.0558
*k*_d0_^O^	prefactor for oxygen desorption	6.0 × 10^13^
*E*_d_^O^	desorption energy of O	2.85
*A*_d_^O^	coverage dependence of the oxygen desorption energy on adsorbed oxygen	–0.400
*B*_d_^O^	coverage dependence of the oxygen desorption energy on molecular oxygen	–0.500
*k*_ox_^0^	prefactor for oxygen diffusion from surface to subsurface sites	9.05 × 10^6^
*k*_red_^0^	prefactor for oxygen diffusion from subsurface to surface sites	7.90 × 10^8^
*A*_red_^s^	subsurface oxygen coverage dependence on surface-subsurface reduction	0.17
*w*_sd_	reduced coupling factor at 433 K due to the step-doubling reconstruction	0.001
*w*_mr_	reduced coupling factor at 433 K due to the missing-row reconstruction	0.650

a Energies are given in eV, rate constants are given in 1/s, and *k*_dif0_^h^ are given in nm^2^/s.

The rate of subsurface oxygen formation and thus the local natural oscillation frequency are determined by the activation energy *E*_ox_ for the formation of subsurface oxygen,^18,20−22^ which is related to the surface roughness, that is, to the crystallographic orientation of the Rh surface.^37^ Accordingly, an *E*_ox_^*i*^ value was assigned to each oscillator of the model network, ranging from 0.700 to 0.769 eV (Figure 8a,c, Table 2), depending on the crystallographic region it represents. Because the local atomic roughness of the surface strongly depends on the curvature of the crystal, the used values should be treated as effective *E*_ox_ values, which may differ from those of planar macroscopic single crystals. The effective activation energy *E*_red_^*i*^ for subsurface oxygen reduction can be directly inferred from the respective *E*_ox_^*i*^ values by the relation^21^
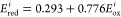


**Table 2:** Local Effective Activation Energies for Subsurface Oxygen Formation (*E*_ox_^*i*^) and Reduction (*E*_red_^*i*^) and Local Initial Oxygen Sticking Coefficients (*S*_0_^O^) Based on the Surface {*hkl*}^a^

{*hkl*}	*E*_ox_^*i*^	*E*_red_^*i*^	*S*_0_^O^
{001}	0.745	0.872	0.9500
{001}_ν_	0.740	0.868	0.9000
{013}	0.728	0.859	0.6620
{115}	0.732	0.862	0.6420
{137}	0.705	0.841	0.5800
{012}	0.713	0.847	0.5800
{113}	0.711	0.846	0.5900
{135}	0.700	0.837	0.5900
{023}	0.701	0.838	0.5670
{236}	0.701	0.838	0.5850
{112}	0.710	0.845	0.5850
{156}	0.713	0.847	0.5740
{357}	0.700	0.837	0.5690
{355}	0.706	0.842	0.5900
{133}	0.704	0.840	0.5900
{011}	0.709	0.844	0.5900
{111}_ν_	0.754	0.879	0.5858
(111)	0.766	0.888	0.5861
(11̅1)	0.769	0.891	0.5861
(11̅1̅)	0.763	0.886	0.5861
(111̅)	0.760	0.884	0.5861

a Energies are given in eV.

The initial sticking coefficient of oxygen *S*_0_^O,*i*^ also depends on the local surface structure, therefore its particular value was assigned for each oscillator, for example, *S*_0_^O,*i*^on the (001) facet was assumed to be 0.95, while *S*_0_^O^ of around 0.6 was used for (111) surfaces.^48^ With increasing step density, the *S*_0_^O,*i*^ of surfaces with (001) terraces were assumed to be decreasing toward 0.6. For the four {111} facets, slightly different *E*_ox_^*i*^ values were assumed, due to their slightly different size. The local values of *E*_ox_^*i*^ and *S*_0_^O,*i*^ for each type of facet and the reduced coupling factors *w*_*i*,*j*_ across the reconstruction-induced diffusion barriers were refined until the experimentally observed phenomena were reproduced in the simulations. The *E*_ox_^*i*^, *E*_red_^*i*^, and *S*_0_^O^ values for all oscillators are given in Table 2.

**Figure 9:**
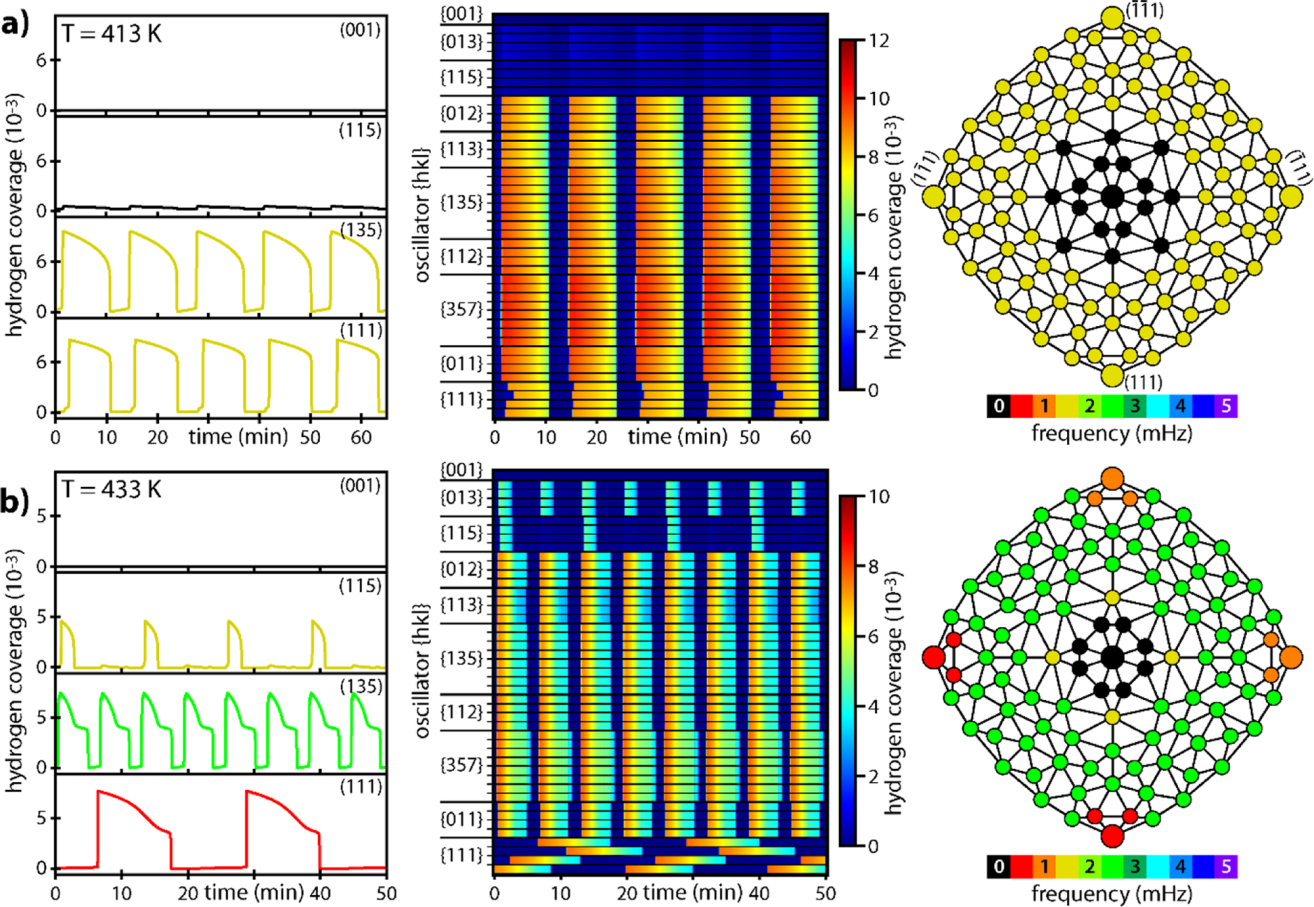
Micro-kinetic modeling of oscillations at constant *p*_H_2__ = 4.8 × 10^–6^ mbar and *p*_O_2__ = 4.4 × 10^–6^ mbar: (a) calculated time series at 413 K for chosen oscillators; middle: the same but as waterfall plot for 45 oscillators; right side: corresponding frequency map, with the oscillator positions corresponding to those shown in Figure 8c; (b) same as in (a), but at 433 K.

The results of the calculations for constant *p*_H_2__ = 4.8 × 10^–6^ mbar and *p*_O_2__ = 4.4 × 10^–6^ mbar are shown in Figure 9a (413 K) and Figure 9b (433 K). At 413 K, the simulations result in monofrequential oscillations synchronized over the entire surface, with exception of the inner circle around the (001) center facet. The left side of Figure 9a shows the time series of the simulated hydrogen coverages for the same {*hkl*} regions, for which the ROIs in the experimental observations were chosen. A bigger set of time series for 45 of the 105 facets is presented in the waterfall plot in the middle of Figure 9a, illustrating the high degree of spatial synchronization. From the calculated time series, the frequency map can be constructed (right side of Figure 9a). The temporal behavior of the oscillators, the local frequencies, and the spatial synchronization mirror well the experimental observations at 413 K (cf. Figure 3).

At 433 K, the model calculations generate multifrequential oscillations: four {111} regions which are decoupled due to reconstruction, oscillate with slightly different frequencies. The ring of facets around the stepped {001} regions is, like at 413 K, internally synchronized with each other in their oscillation behavior. The {013} regions, nonoscillating at 413 K, oscillate at 433 K, while the {115} regions display oscillations with a double period compared to those of the adjacent oscillators. All results presented in Figure 9 fit the experimental behavior (Figures 3 and 4) very well, for example, the temperature-dependent frequency of kinetic oscillations (cf. Figure 9a,b), which results from the temperature-dependent and reconstruction-modified rate of subsurface oxygen formation.

The explanation of the occurring phenomena can be traced back to the interfacet coupling via hydrogen diffusion. Due to the barrier-like effects of the local step-doubling reconstructions on the vicinal {111} regions, the communication between the “ring” region of the surface and the enclosed {111} regions decreases; therefore, these crystallographically different regions oscillate independently from their neighboring facets, which results in multifrequential oscillations. As the size of the four {111} facets slightly varies, the structure of the local step and kink geometry differs. The resulting small variations in the respective *E*_ox_^*i*^ are responsible for the minor deviations of the local oscillation frequencies observed in the experiments (Figure 5) and in the model simulations (Figure 9b).

For the {115} facets, two processes, the hindered diffusive supply of hydrogen resulting from the missing-row reconstruction and the relatively slow formation and depletion of subsurface oxygen, lead to a situation, in which the available amount of hydrogen is only sufficient to induce a transition to the active state every second oscillation cycle of its neighbors. Again, this fits very well with the experimental behavior and can explain the period doubling observed for the {115} facets in the experiment (cf. Figure 4b). Generally, the present model simulations corroborate well our assumption that the reconstruction-induced collapse of spatial coupling is responsible for the emergence of multifrequential oscillations between 413 and 433 K*.*

## Summary

The present experimental and modeling results demonstrate how the appearance of a few atomic rows and nm-sized double-atomic step regions on the surface of an individual Rh particle can “revolutionize” its catalytic behavior. This highlights the essential role of solitary atomic size surface features in the catalytic performance of the entire submicrometer or even much bigger particles, as observed earlier for atomic rows along the metal/oxide boundaries.^53^ The key to the discovery of the present effects was choosing the proper size of the specimen: on the one hand, the size of the “pacemaker nanofacets”^10^ must be large enough for distinctive surface reconstruction, while on the other hand, the curvature radius must be small enough for FEM imaging. The curved Rh crystal with a radius of 650 nm used in the present study fulfills both prerequisites.

This way, the essential role of spatial coupling for the behavior of the entire particle is elucidated: in the monofrequential oscillation mode of H_2_ oxidation, almost the whole surface behaves synchronously, although differently oriented nanofacets should, in principle, exhibit different frequencies, for example, as different domains of a polycrystal indeed do.^20,21^ The expected “natural” frequencies of individual regions are governed by the rate of subsurface oxygen formation/depletion, which is structure dependent.^22,37^ Consequently, in the present case many differently oriented facets of the Rh particle oscillate with a “forced” frequency, other than their “natural” frequency. The heterogeneous surface, thus, behaves as an extended system of diffusively coupled oscillators, for which known effects such as entrainment and frequency-locking may occur.^54,55^ Both effects are related to periodic forcing, a phenomenon that is ubiquitous in nature and can be observed in human and animal heartbeats, electronic circuits, economics, and other systems.^56−59^ Apparently, we observed a similar phenomenon in catalysis on the nanoscale: every unicolor region in the frequency maps in Figure 4 represents frequency-locked uniform oscillations with a frequency corresponding to the natural frequency of a specific {*hkl*}-orientation and with the rest of the surface “joining in”. The microkinetic modeling using a network of 105 coupled individual oscillators explains both, the synchronized behavior, and the loss of synchronization upon weakening of the coupling chain links.

Clearly, particles of technological catalysts also have certain facets of particular {*hkl*}-orientation, which may serve as coupled pacemakers of catalytic activity. Their identification and revealing of the role of coupling is of significant interest but remains a great challenge at this time. Future nm-scale studies at higher pressures for this and other reactions on model systems mimicking single particles hold promise for more discoveries of nanofacet reactivity and interfacet communication effects.

## References

[ref1] Hansen T. W.; Wagner J. B.; Hansen P. L.; Dahl S.; Topsøe H.; Jacobsen C. J. H. Atomic-Resolution in Situ Transmission Electron Microscopy of a Promoter of a Heterogeneous Catalyst. Science 2001, 294, 1508–1510. 10.1126/science.1064399. 11711670

[ref2] Liu Y.; Meirer F.; Krest C. M.; Webb S.; Weckhuysen B. M. Relating structure and composition with accessibility of a single catalyst particle using correlative 3-dimensional micro-spectroscopy. Nat. Commun. 2016, 7, 12634 10.1038/ncomms12634. 27572475 PMC5013607

[ref3] Kim Y. Y.; Keller T. F.; Goncalves T. J.; Abuin M.; Runge H.; Gelisio L.; Carnis J.; Vonk V.; Plessow P. N.; Vartaniants I. V.; Stierle A. Single alloy nanoparticle x-ray imaging during a catalytic reaction. Sci. Adv. 2021, 7, eabh0757 10.1126/sciadv.abh0757. 34597137 PMC10938497

[ref4] Dery S.; Amit E.; Gross E. Identifying Catalytic Reactions on Single Nanoparticles. Top. Catal. 2018, 61, 923–939. 10.1007/s11244-018-0931-4.

[ref5] Suchorski Y. Field Ion and Field Desorption Microscopy: Surface Chemistry Applications. In Encyclopedia of Interfacial Chemistry-Surface Science and Electrochemistry; Wandelt K., Ed.; Elsevier Inc., 2018; pp 162–179.

[ref6] Gorodetskii V.; Drachsel W.; Block J. H. Imaging the oscillating CO-oxidation on Pt-surfaces with field ion microscopy. Catal. Lett. 1993, 19, 223–231. 10.1007/bf00771758.

[ref7] Chau T.-D.; Visart de Bocarmé T.; Kruse N. Kinetic instabilities in the NO/H_2_ reaction on platinum. Surf. Interface Anal. 2004, 36, 528–532. 10.1002/sia.1701.

[ref8] Visart de Bocarmé T.; Bär T.; Kruse N. In situ dynamic study of hydrogen oxidation on rhodium. Ultramicroscopy 2001, 89, 75–82. 10.1016/s0304-3991(01)00114-0. 11770755

[ref9] Suchorski Y.; Bespalov I.; Zeininger J.; Raab M.; Datler M.; Winkler P.; Rupprechter G. CO oxidation on stepped Rh surfaces: μm-scale versus nanoscale. Catal. Lett. 2020, 150, 605–612. 10.1007/s10562-019-02950-0. PMC7175702 32355436

[ref10] Zeininger J.; Suchorski Y.; Raab M.; Buhr S.; Grönbeck H.; Rupprechter G. Single particle catalysis: revealing intraparticle pacemakers in catalytic H_2_ oxidation on Rh. ACS Catal. 2021, 11, 10020–10027. 10.1021/acscatal.1c02384. 34386273 PMC8353627

[ref11] Medvedev V. K.; Suchorski Y.; Block J. H. Oscillations of the CO oxidation on Rh induced by field-controlled Li coadsorption. Surf. Sci. 1995, 343, 169–175. 10.1016/0039-6028(95)00694-x.

[ref12] Medvedev V. K.; Suchorski Y.; Block J. H. Li-mediated feedback mechanism of oscillations in CO oxidation on a Rh field emitter tip. Appl. Surf. Sci. 1996, 94–95, 200–206. 10.1016/0169-4332(95)00376-2.

[ref13] Suchorski Y. Probing adsorption on a nanoscale: field desorption microspectroscopy. Adsorption 2017, 23, 217–224. 10.1007/s10450-016-9824-7. 32214678 PMC7064037

[ref14] Beck A.; Huang X.; Artiglia L.; Zabilskiy M.; Wang X.; Rzepka P.; Palagin D.; Willinger M.-G.; van Bokhoven J. A. The dynamics of overlayer formation on catalyst nanoparticles and strong metal-support interaction. Nat. Commun. 2020, 11, 3220 10.1038/s41467-020-17070-2. 32591532 PMC7320156

[ref15] Chee S. W.; Lunkenbein T.; Schlögl R.; Cuenya B. In situ and operando electron microscopy in heterogeneous catalysis insights into multi-scale chemical dynamics. J. Phys.: Condens. Matter 2021, 33, 153001 10.1088/1361-648x/abddfd. 33825698

[ref16] Shi J.; Li H.; Genest A.; Zhao W.; Qi P.; Wang T.; Rupprechter G. High-performance water gas shift induced by asymmetric oxygen vacancies: Gold clusters supported by ceria-praseodymia mixed oxides. Appl. Catal., B 2022, 301, 120789 10.1016/j.apcatb.2021.120789.

[ref17] Gorodetskii V.; Lauterbach J.; Rotermund H. H.; Block J. H.; Ertl G. Coupling between adjacent crystal planes in heterogeneous catalysis by propagating reaction–diffusion waves. Nature 1994, 370, 276–279. 10.1038/370276a0.

[ref18] Suchorski Y.; Zeininger J.; Buhr S.; Raab M.; Stöger-Pollach M.; Bernardi J.; Grönbeck H.; Rupprechter G. Resolving multifrequential oscillations and nanoscale interfacet communication in single particle catalysis. Science 2021, 372, 1314–1318. 10.1126/science.abf8107. 34016741

[ref19] Suchorski Y.; Rupprechter G. Local Reaction Kinetics by Imaging. Surf. Sci. 2016, 643, 52–58. 10.1016/j.susc.2015.05.021. 26865736 PMC4705865

[ref20] Suchorski Y.; Datler M.; Bespalov I.; Zeininger J.; Stöger-Pollach M.; Bernardi J.; Grönbeck H.; Rupprechter G. Visualizing catalyst heterogeneity by a multifrequential oscillating reaction. Nat. Commun. 2018, 9, 600 10.1038/s41467-018-03007-3. 29426883 PMC5807506

[ref21] Suchorski Y.; Datler M.; Bespalov I.; Zeininger J.; Stöger-Pollach M.; Bernardi J.; Grönbeck H.; Rupprechter G. Surface-Structure Libraries: Multifrequential Oscillations in Catalytic Hydrogen Oxidation on Rhodium. J. Phys. Chem. C 2019, 123, 4217–4227. 10.1021/acs.jpcc.8b11421. PMC6494118 31057690

[ref22] Winkler P.; Zeininger J.; Raab M.; Suchorski Y.; Steiger-Thirsfeld A.; Stöger-Pollach M.; Amati M.; Gregoratti L.; Grönbeck G.; Rupprechter G. Coexisting multi-states in catalytic hydrogen oxidation on rhodium. Nat. Commun. 2021, 12, 6517 10.1038/s41467-021-26855-y. 34764290 PMC8586342

[ref23] Suchorski Y. Surface Diffusion Via Adsorbate Density Fluctuations. In Encyclopedia of Interfacial Chemistry-Surface Science and Electrochemistry; Wandelt K., Ed.; Elsevier Inc., 2018; pp 648–665.

[ref24] Suchorski Y.; Rupprechter G. Catalysis by imaging: from meso- to nano-scale. Top. Catal. 2020, 63, 1532–1544. 10.1007/s11244-020-01302-2.

[ref25] Gorodetskii V. V.; Nieuwenhuys B. E. Chemisorption and dissociation of carbon monoxide on rhodium surfaces. Surf. Sci. 1981, 105, 299–312. 10.1016/0039-6028(81)90163-1.

[ref26] Gregoratti L.; Baraldi A.; Dhanak V. R.; Comelli G.; Kiskinova M.; Rosei R. Structural effects on water formation from coadsorbed H+O on Rh(100). Surf. Sci. 1995, 340, 205–214. 10.1016/0039-6028(95)00695-8.

[ref27] Datler M.; Bespalov I.; Buhr S.; Zeininger J.; Stöger-Pollach M.; Bernardi J.; Rupprechter G.; Suchorski Y. Hydrogen oxidation on stepped Rh surfaces: μm-scale versus nanoscale. Catal. Lett. 2016, 146, 1867–1874. 10.1007/s10562-016-1824-4. PMC7175702 32355436

[ref28] Zuniga G. E.; Luss D. Kinetic Oscillations during the Isothermal Oxidation of Hydrogen on Platinum Wires. J. Catal. 1978, 53, 312–320. 10.1016/0021-9517(78)90103-3.

[ref29] Lalik E.; Drelinkiewicz A.; Kosydar R.; Szumełda T.; Bielańska E.; Groszek D.; Iannetelli A.; Groszek M. Oscillatory Behavior and Anomalous Heat Evolution in Recombination of H_2_ and O_2_ on Pd-based Catalysts. Ind. Eng. Chem. Res. 2015, 54, 7047–7058. 10.1021/acs.iecr.5b00686.

[ref30] Imbihl R. Nonlinear dynamics on catalytic surfaces: The contribution of surface science. Surf. Sci. 2009, 603, 1671–1679. 10.1016/j.susc.2008.11.042.

[ref31] Ertl G. Reactions at surfaces: From atoms to complexity (Nobel Lecture). Angew. Chem., Int. Ed. 2008, 47, 3524–3535. 10.1002/anie.200800480. 18357601

[ref32] Slinko M.; Fink T.; Löher T.; Madden H. H.; Lombardo S. J.; Imbihl R.; Ertl G. The NO + H_2_ reaction on Pt(100): steady state and oscillatory kinetics. Surf. Sci. 1992, 264, 157–170. 10.1016/0039-6028(92)90174-5.

[ref33] Yu W.; Yu X.; Tu S.-T. Oxidation of hydrogen off-gas from a fuel cell using a microstructured reactor with hydrophobic Pt-Al_2_O_3_ catalyst coating. Energy Proc. 2014, 61, 2854–2857. 10.1016/j.egypro.2014.12.322.

[ref34] Saint-Just J.; Etemad S. Catalytic combustion of hydrogen for heat production. In Compendium of Hydrogen Energy Volume 3: Hydrogen Energy Conversion; Babir F., Basile A., Veziroğlu T. N., Eds.; Elsevier Inc., Woodhead Publishing, 2016; pp 263–287.

[ref35] Steffen P. M.; Reinecke E. A.; Meynet N.; Bentaib A.; Chaumeix N.; Allelein H. J. Operational behavior of a passive auto-catalytic recombiner under low pressure conditions. Fusion Eng. Des. 2017, 124, 1281–1286. 10.1016/j.fusengdes.2017.02.019.

[ref36] Hübert T.; Boon-Brett L.; Black G.; Banach U. Hydrogen sensors – A review. Sens. Actuators, B 2011, 157, 329–352. 10.1016/j.snb.2011.04.070.

[ref37] Winkler P.; Zeininger J.; Suchorski Y.; Stöger-Pollach M.; Zeller P.; Amati M.; Gregoratti L.; Rupprechter G. How the anisotropy of surface oxide formation influences the transient activity of a surface reaction. Nat. Commun. 2021, 12, 69 10.1038/s41467-020-20377-9. 33398022 PMC7782819

[ref38] Pikovsky A.; Rosenblum M.; Kurths J. Synchronization. A Universal Concept in Nonlinear Sciences; Cambridge University Press, 2018; pp 49–68.

[ref39] Eiswirth M.; Krischer K.; Ertl G. Transition to chaos in an oscillating surface reaction. Surf. Sci. 1988, 202, 565–591. 10.1016/0039-6028(88)90053-2.

[ref40] Murray P. W.; Leibsle F. M.; Li Y.; Guo Q.; Bowker M.; Thornton G.; Dhanak V. R.; Prince K. C.; Rosei R. Scanning-tunneling-microscopy study of the oxygen-induced reconstruction of Rh(110). Phys. Rev. B: Condens. Matter Mater. Phys. 1993, 47, 12976–12979. 10.1103/physrevb.47.12976. 10005505

[ref41] Voss C.; Gaussmann A.; Kruse N. Oxygen-induced reconstruction of Rh{110} and {113} single crystal planes. Appl. Surf. Sci. 1993, 67, 142–146. 10.1016/0169-4332(93)90306-v.

[ref42] Medvedev V. K.; Suchorski Y.; Voss C.; Visart de Bocarmé T.; Bär T.; Kruse N. Oxygen-induced reconstruction and surface oxidation of rhodium. Langmuir 1998, 14, 6151–6157. 10.1021/la980603c.

[ref43] Hoogers G.; King D. A. Adsorbate-induced step-doubling reconstruction of a vicinal metal surface: oxygen on Rh {332}. Surf. Sci. 1993, 286, 306–316. 10.1016/0039-6028(93)90414-f.

[ref44] Mertens F.; Imbihl R.; Mikhailov A. Breakdown of global coupling in oscillatory chemical reactions. J. Chem. Phys. 1993, 99, 8668–8671. 10.1063/1.465590.

[ref45] McEwen J.-S.; Gaspard P.; Visart de Bocarme T.; Kruse N. Oscillations and bistability in the catalytic formation of water on rhodium in high electric fields. J. Phys. Chem. C 2009, 113, 17045–17058. 10.1021/jp901975w.

[ref46] Suchorski Y.; Imbihl R.; Medvedev V. K. Compatibility of field emitter studies of oscillating surface reactions with single crystal measurements: Catalytic CO oxidation on Pt. Surf. Sci. 1998, 401, 392 10.1016/s0039-6028(98)00043-0.

[ref47] Imbihl R.; Ladas S.; Ertl G. Spatial coupling of autonomous kinetic oscillations in the catalytic CO oxidation on Pt(110). Surf. Sci. 1989, 215, L307–L315. 10.1016/0039-6028(89)90260-4.

[ref48] McEwen J.-S.; Gaspard P.; Visart de Bocarmé T.; Kruse N. Electric field induced oscillations in the catalytic water production on rhodium: A theoretical analysis. Surf. Sci. 2010, 604, 1353–1368. 10.1016/j.susc.2010.04.007.

[ref49] Makeev A.; Imbihl R. Simulations of anisotropic front propagation in the H_2_+O_2_ reaction on a Rh(110) surface. J. Phys. Chem. C 2000, 113, 3854–3863. 10.1063/1.1287797.

[ref50] Seebauer E. G.; Kong A. C. F.; Schmidt L. D. Surface diffusion of hydrogen and CO on Rh(111): Laser-induced thermal desorption studies. J. Chem. Phys. 1988, 88, 6597–6604. 10.1063/1.454447.

[ref51] Mann S. S.; Seto T.; Barnes C. J.; King D. A. Coverage dependence of surface diffusion of hydrogen and deuterium on Rh{111) by laser induced thermal desorption. Surf. Sci. 1992, 261, 155–163. 10.1016/0039-6028(92)90227-w.

[ref52] Hoogers G.; Lesiák-Orłowska B.; King D. A. Diffusion on a stepped surface: H and D on Rh{332}. Surf. Sci. 1995, 327, 47–52. 10.1016/0039-6028(94)00825-6.

[ref53] Suchorski Y.; Datler M.; Bespalov I.; Freytag C.; Zeininger J.; Rupprechter G. Transmitting metal-oxide interaction by solitary chemical waves: H_2_ oxidation on ZrO_2_ supported Rh. Surf. Sci. 2019, 679, 163–168. 10.1016/j.susc.2018.08.027.

[ref54] Krefting D.; Kaira P.; Rotermund H. H. Period doubling and spatiotemporal chaos in periodically forced CO oxidation on Pt(110). Phys. Rev. Lett. 2009, 102, 178301 10.1103/physrevlett.102.178301. 19518840

[ref55] Bodega P. S.; Kaira P.; Beta C.; Krefting D.; Bauer D.; Mirwald-Schulz B.; Punckt C.; Rotermund H. H. High frequency periodic forcing of the oscillatory catalytic CO oxidation on Pt (110). New J. Phys. 2007, 9, 61 10.1088/1367-2630/9/3/061.

[ref56] Guerrini L. Hopf Bifurcation analysis of a dynamical heart model with time delay. Appl. Math. Sci. 2017, 11, 1089–1095. 10.12988/ams.2017.7271.

[ref57] Rulkov N. F. Images of synchronized chaos: experiments with circuits. Chaos 1996, 6, 262–279. 10.1063/1.166174. 12780256

[ref58] Van Der Pol B.; Van Der Mark J. Frequency demultiplication. Nature 1927, 120, 363–364. 10.1038/120363a0.

[ref59] Sasakura K. Political economic chaos?. J. Econ. Behav. Organ. 1995, 27, 213–221. 10.1016/0167-2681(94)00080-x.

